# Determination of bisphosphonate properties in terms of bioavailability, bone affinity, and cytotoxicity

**DOI:** 10.1007/s43440-024-00624-2

**Published:** 2024-07-15

**Authors:** Monika Zielińska, Amanda Pacholak, Natalia Burlaga, Ewa Chmielewska, Adam Voelkel, Ewa Kaczorek

**Affiliations:** 1 Institute of Chemical Technology and Engineering, Poznań University of Technology, Berdychowo 4, 60-965 Poznań, Poland; 2 Faculty of Chemistry, Department of Bioorganic Chemistry, Wrocław University of Science and Technology, Wrocław, Poland

**Keywords:** Bisphosphonates, Cytotoxicity, Hydroxyapatite, Artificial membrane permeability

## Abstract

**Background:**

The study aimed to evaluate the therapeutic potential of fourteen newly synthesized bisphosphonates by assessing their bioavailability, bone affinity, and cytotoxicity. These bisphosphonates included a series of aminomethylenebisphosphonates and standard compounds such as risedronate and tiludronate.

**Methods:**

Drug permeability was determined using Parallel Artificial Membrane Permeability Assays (PAMPA), while bone affinity was assessed by sorption on hydroxyapatite. Bacterial cell response to the bisphosphonates was also examined using *Lactobacillus paracasei* cells as a model.

**Results:**

Several tested compounds, including BP3 to BP8 and BP11, which feature substituents in the pyridine ring such as methyl groups, iodine, bromine, chlorine, or hydroxyl groups, demonstrated potentially more beneficial therapeutic properties than commercially used bisphosphonates. These compounds showed stronger bone affinity and higher gastrointestinal absorption with comparable or lower cytotoxic effects. Specifically, BP11 exhibited the highest bone affinity, while BP8 and BP11 showed the greatest permeability.

**Conclusions:**

The findings suggest that BP3 BP8, and BP11 are promising candidates for further research. These results highlight the importance of comprehensively evaluating bisphosphonates' therapeutic properties to identify effective treatments for osteoporosis and other bone diseases.

**Graphical abstract:**

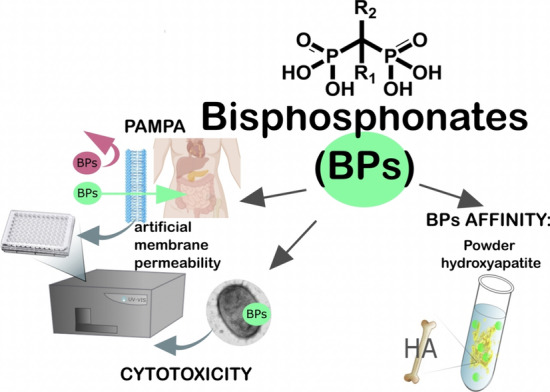

## Introduction

Bisphosphonates (BPs) are used to treat various bone diseases such as osteoporosis, Paget's disease, multiple myeloma, and others [1]. Osteoporosis affects the human skeletal system by causing a decrease in bone density and disturbances in the integrity of their tissues. In the course of this disease, there is a decrease in bone density and disturbances in the integrity of their tissues, which results in a much higher susceptibility to fractures [2]. BPs have a high affinity for hydroxyapatite (HA) due to their ability to bind calcium ions, therefore BPs rapidly accumulate in the bone where they inhibit the activity of bone-destroying osteoclasts [3]. However, the affinity for HA varies depending on the structure of the BPs. The diverse biological activity seen in BPs is primarily attributed to the two side chains (R1 and R2) connected to the carbon atom. Particularly, R1 substituents, including hydroxyl or amino groups, are crucial in improving the chemical binding to minerals [4]. The critical features of the structure–activity relationships for the interactions between BPs and HA show that nitrogen moiety, and its position in the heterocyclic ring or alkyl group in the side chain (R2), can lead to significant increases in HA binding. For maximal potency, the nitrogen atom in the side chain must be a critical distance away from the P-C-P group, and in a specific spatial configuration [4, 5].

BPs will show their antiresorptive properties only when absorbed from the gastrointestinal tract (if oral administration is applied). Their oral bioavailability is low, reaching one percent. For example, risedronate (RSD), a commonly used aminobisphosphonate, bioavailability is 0.62% [6]. The low level of absorption is due to high affinity for food (especially food rich in divalent cations) and their short absorption window in the duodenum. Moreover, BPs are assigned to the III class Biopharmaceutics Classification System (BCS) and occur as well-soluble in water but poorly absorbable substances. The poor absorption is attributed to their very poor lipophilicity, which prevents transcellular transport across the epithelial barriers [7]. The development of additional lipophilic compounds appears to be a worthwhile goal because membrane transport plays an important role [8]. In vitro*,* artificial intestinal membrane permeability study was used to determine the degree of zoledronic acid absorption with Caco-2 cell monolayer [9]. In this work of Jeon O-C et al., an attempt was made to assess the permeability *P*_*e*_ of zoledronate but no satisfactory results were obtained and the result was "Not detected". However, the apparent permeability coefficient *P*_*app*_ was tested using a Caco-2 cell monolayer. The *P*_*app*_ for zoledronate was then 2.72 ± 0.868 (× 10^−6^) cm s^−1^. The authors also studied ionic complexes by electrostatic conjugation of zoledronic acid with lysine-linked deoxycholic acid (Lys-DOCA). In vitro, permeabilities of zoledronic acid/Lys-DOCA was *P*_*e*_ 0.250 ± 0.242 (× 10^−6^) cm s^−1^ and *P*_*app*_ was 6.72 ± 1.94 (× 10^−6^) cm s^−1^. It can be concluded that the permeability *P*_*e*_ of zoledronate did not exceed the detection limit. Similar studies with parallel artificial membrane permeability assay (PAMPA) will be carried out in this work. PAMPA is more automated and allows for rapid permeability measurements, whereas the Caco-2 test uses live intestinal cells, reflecting more natural physiological conditions in substance permeability assessment. PAMPA first introduced by Kansy et al. [10] was widely implemented in the pharmaceutical industry to predict oral absorption as a high throughput permeability assay. Compounds that have a permeability < 10 × 10^−6^ cm s^−1^ are classified as low permeability and compounds with a permeability > 10 × 10^−6^ cm s^−1^ are classified as high permeability [11]. PAMPA allows for quicker and less costly permeability measurements compared to e.g. Caco-2. This reason for use was given by other authors who applied PAMPA for ibandronate and ibandronate with gluco- or galactopyranoside derivatives analysis [12].

BPs have low bioavailability (about 1% of nitrogen-containing BPs when taken orally) but afterward, only 50% of the absorbed drug undergoes selective retention in the skeletal system, while the rest is removed by the kidneys in an unchanged form. Since the BPs are not fully absorbed and retained in the skeleton, they are expected to be excreted in the urine. Therefore, the 24-h excretion of BPs after a single dose has been used as an inverse measure of the amount retained by the skeleton [13]. On this basis, the BPs were ranked similarly to their mineral affinity (an inverse order compared with their mineral binding affinities). The rank order of urinary excretion 24 h after the first dose was zoledronate (38%) < alendronate (44%) < risedronate (65%) < clodronate (73%), as expected [4]. BPs with a strong affinity for HA are sought. In our previous work [14], several BPs with more favourable properties, than the standardly used RSD, were selected. Promising BPs were decided to undergo further research. However, the tests were conducted for low concentrations of drugs. Sorption of BPs on 30 mg of HA was carried out with 3 mL of SBF solution containing BPs (0.164 mM). Almost 100% sorption was then achieved for 12 tested BPs. The research in this work will be continued at a higher BP concentration and higher sample volume. Hydrophilicity and the n-octanol-water partition coefficient (log P) play crucial roles in determining the behaviour of BPs in the body. BPs are classified based on their hydrophilic or lipophilic nature, which influences their absorption and distribution. Lipophilic BPs are noted to exhibit higher activity compared to hydrophilic ones, as indicated in studies such as Zhang et al. [15]. The negative log p values for hydrophilic substances suggest limited diffusion across biological membranes. For instance, zoledronate, known for its highly polar characteristics (log p = − 2.9), demonstrated poor permeability in specific assays [16]. This highlights the significance of hydrophilicity and log P values in understanding the pharmacological properties and bioavailability of BPs.

Orally administered BPs can cause a variety of side effects, including an upset stomach, as well as inflammation and erosions in the esophagus. Nausea, vomiting, and diarrhea may also occur [1, 2, 17, 18]. Therefore, the BP effect on the beneficial bacterial cells, colonizing the human stomach cannot be neglected. While the human gut is colonized by various bacteria, with higher concentrations in the colon compared to the stomach, the impact of BPs on lactic acid bacteria remains relevant due to their presence and functional significance in the gastrointestinal tract. For this reason, cytotoxicity studies were performed in this work. Recently, efforts have been made to enhance the viability of beneficial lactic acid bacteria in the human gastrointestinal tract [19–21]. Recent research has also focused on the determination of the impact of orally administered pharmaceuticals on lactic acid bacteria, colonizing the human gut [22–24]. Therefore, the impact of newly synthesized drugs on these bacteria should be thoroughly examined.

The aim of this study was to select bisphosphonates, with more favorable properties than those commercially available, based on three parameters: bioavailability based on the intestinal absorption through the parallel artificial membrane permeability assay (PAMPA), BP affinity to HA- the main component of bone, cytotoxicity—effect of the exposure of twelve BPs on *Lactobacillus paracasei* cells – their surface properties, growth rate, and metabolic activity.

## Materials and methods

### Materials

Hydroxyapatite (HA) purity p.a., ≥ 90% (average particle size 13 μm) 21,223 (cat number), sodium risedronate (RSD) 1,604,610, tiludronate disodium salt hydrate (TIL) T4580 and tris(hydroxymethyl)aminomethane (998%) (TRIS) T1503, Phosphate Buffered Saline (PBS) were obtained from Sigma-Aldrich (Steinheim, Germany) D5652. Methanol p.a. 621,991,154, hydrochloric acid (36–38%) 575,283,115, and sodium chloride (99%) 794,121,116 were obtained from POCh (Gliwice, Poland). Culture media (MRS Agar, CM0361B, and MRS Broth, CM0359B) were obtained from Oxoid™, Thermo Scientific™ (Waltham, MA, USA). Each solution was prepared using ultrapure water (Arium® Pro Ultrapure Water System, Sartorius, Göttingen, Germany). The aqueous solutions and glassware were steam sterilized before using them in the experiments (HG80 Autoclave, HMC-Europe, Tüssling Germany). Non-autoclavable solutions were filter sterilized (0.22 μm). The biological safety cabinet (Labculture® Class II, Esco, Singapure) was used to prevent sample contamination.

Twelve aminomethylenebisphosphonates used in this study (Table 1) were prepared according to the procedure described previously [14].

**Table 1:** Structures of bisphosphonates (BPs) used in this study

No	Compound name	Compound structure	References
RSD	Risedronate		[4]
TIL	Tiludronate		[4]
BP1	2-pyridinylaminomethylene-1,1-bisphosphonic acid		[25]
BP 2	(3-methyl-2-pyridinyl)aminomethylene-1,1-bisphosphonic acid		[26]
BP 3	(4-methyl-2-pyridinyl)aminomethylene-1,1-bisphosphonic acid		[25]
BP 4	(5-methyl-2-pyridinyl)aminomethylene-1,1-bisphosphonic acid		[26]
BP 5	(4,6-dimethyl-2-pyridinyl)amino-methylene-1,1-bisphosphonic acid		[8, 14]
BP 6	(5-iodo-2-pyridinyl)amino-methylene-1,1-bisphosphonic acid		[8, 14]
BP 7	(5-bromo-2-pyridinyl)amino-methylene-1,1-bisphosphonic acid		[27]
BP 8	(5-chloro-2-pyridinyl)amino-methylene-1,1-bisphosphonic acid		[26]
BP 9	(6-chloro-2-pyridinyl)amino-methylene-1,1-bisphosphonic acid		[14]
BP 10	2-chloro-3-pyridinyl)amino-methylene-1,1-bisphosphonic acid		[28]
BP 11	(6-hydroksy-2-pyridinyl)amino-methylene-1,1-bisphosphonic acid		[14]
BP 12	*N*-(1,3-benzothiazol-2-yl)amino-methylene-1,1-bisphosphonic acid		[29]

### A parallel artificial membrane permeability assay (PAMPA)

A parallel artificial membrane permeability assay (PAMPA) (Corning® Gentest™ Pre-coated PAMPA Plate System Gentest, USA) was prepared according to the recommended protocol. 96-well PAMPA plate was heated to room temperature for at least 30 min. Compound solutions were prepared in buffer (175 μM in PBS, pH 7.4). 300 μL of the BP solution was added to each donor plate well, and 200 μL of buffer (PBS, pH 7.4) was added to each acceptor plate. The filter plate (acceptor plate) was then placed on the receiver plate (donor plane) and the plate assembly was incubated at room temperature for 5 h. The compound amounts were measured by Multiskan152 SkyMicroplate Spectrophotometer (Thermo Fisher Scientific, Waltham, USA). The permeability of the BPs was calculated using the following formula (Eq. 1)

1 $$P_e = \frac{{ - In[1 - \frac{C_A (t)}{{C_{equilibrium} }}]}}{A*(1/V_D + 1/V_A )*t}$$

where P_e_ is the permeability (cm s^−1^), C_equilibrium_ is [C_D_(t) × V_D_ + C_A_(t) × V_A_]/(V_D_ + V_A_), C_D_(t) indicates the compound concentration in donor well at time t (mM), C_A_(t) indicates the compound concentration in acceptor well at time t (mM), V_D_ is the donor well volume (0.3 mL), V_A_ is the acceptor well volume (0.2 mL), t is the incubation time (18000 s), and A is the effective filter area (0.3 cm^2^).

### Bisphosphonates (BPs) affinity

The first step was to investigate the effect of concentration on the RSD sorption on HA. The sorption capacity was examined for 4 concentrations (0.2, 0.5, 0.7 and 1 mM). 30 mg (+ /− 0.1 mg) of HA was weighed into polypropylene tubes. Subsequently, 10 mL of Simulated Body Fluid (SBF) (Table 2) containing BPs was poured into the tubes and placed in a shaker for 1 h, 2 h, and 48 h. Sorption of BP compounds on HA was measured using a UV–VIS spectrophotometer after 5 min of centrifugation (speed 4000 rpm). The compound concentrations were measured by Multiskan152 Sky Microplate Spectrophotometer (Thermo Fisher Scientific, Waltham, MA, USA). Next, for one concentration (0.7 mM), sorption studies of all BPs were conducted in the same manner as indicated above for RSD. The measurements were conducted in the range of 220–400 nm. The quantitative assessment was performed based on the calibration curve method at the absorbance maximum for each BP (absorption properties of the tested compounds are summarized in Table 3).

**Table 2:** SBF concentration (1000 mL of the SBF) [30]

Order	Reagent	Amount
1	NaCl	8.035 g
2	NaHCO_3_	0.355 g
3	KCl	0.225 g
4	K_2_HPO_4_·3H_2_O	0.231 g
5	Na_2_SO_4_	0.072 g
6	TRIS	0.6112 g
7	HCl	0–5 mL

**Table 3:** Absorption properties of the tested compounds

Compound	λ_max_	ε_max_	λ_max_	ε_max_
RSD	262	3143		
TIL	266	11,017		
BP1	244	11,646	316	4320
BP2	243	10,480	311	5817
BP3	246	9166	310.5	4331
BP4	246	13,611	323	5371
BP5	246	9097	316	6046
BP6	254	14,526	326	1257
BP7	256	15,623	323	2594
BP8	254	17,954	324	2949
BP9	251	13,634	314	4297
BP10	256	12,857	317	2914
BP11	242	16,229	344	5566
BP12	224	21,680	280	11,463

### Cytotoxicity

#### Bacterial strains and growing conditions

*Lactobacillus paracasei* PCM 3039 used in the experiments was purchased from the Polish Collection of Microorganisms (Wroclaw, Poland). The liquid cultures were composed of the minimal medium (10% solution of MRS Broth in Dulbecco’s Phosphate Buffered Saline (DPBS)), the inoculum, and a BP stock solution in water (initial concentration of BPs in each culture was 0.2 mg mL^−1^). The bacterial inoculum was prepared from the broth cultures incubated overnight at 37 °C and washed twice with a sterile medium. The initial cell concentration in each culture was adjusted to 1–10^8^ cfu mL^−1^. The reference cultures without the addition of BPs (denoted as Ctrl) and the abiotic control samples with the addition of pure minimal medium instead of bacterial inoculum were carried out. All samples were incubated at 36 °C for 24 h with shaking (150 rpm).

#### Growth curves and cell properties measurements

The growth of cells treated with BPs as well as control cells was monitored by measuring the optical density at the wavelength of 600 nm (OD_600_) as described by [31]. The growth curve measurements were performed with a microplate reader Multiskan 152 Sky (Thermo Fisher Scientific, Waltham, MA, USA). 200 μL of the prepared bacterial cultures were transferred to the 96-well clear bottom sterile microplates wells. The plates were maintained at 37 °C with pulse shaking. The OD_600_ of each well was read every 10 min for 24 h.

The effect of BPs on the properties of bacterial cells was also evaluated. The experiments performed include (i) modifications of the bacterial cell membrane permeability—the measurements were based on the Crystal Violet uptake by the cells [32], (ii) modifications of the bacterial cell wall hydrophobicity, based on the procedure described previously [32]. Relative metabolic activity of cells treated with the compounds in question measured with the colorimetric test AlamarBlue® Cell Viability Assay [33].

### Statistical analysis

The bioavailability and bone affinity experiments have been performed as five independent repetitions and analyzed with two-way ANOVA followed by Tukey's test for multiple comparisons with a 5% significance level. For the PAMPA permeability results, a one-way ANOVA followed by Dunnett's test for multiple comparisons with a 5% significance level was used. The calculations were performed using GraphPad Prism v10 (GraphPad Software, LLC, San Diego, CA, USA).

The results of the microbiological experiments have been obtained from six measurements (three independent biological replicates and two technical repetitions for each biological replicate). The results were analyzed with a one-way ANOVA followed by Tukey's test for multiple comparisons with a 5% significance level. In the box and whiskers plots, whiskers represent the 10th to 90th percentiles, the box extends from the 25th to 75th percentiles, the midline is plotted at the median, and “ + ” is at the mean value. The calculations were performed using GraphPad Prism v10 (GraphPad Software, LLC, San Diego, CA, USA).

## Results

### Sorption on hydroxyapatite (HA)

**Fig. 1:**
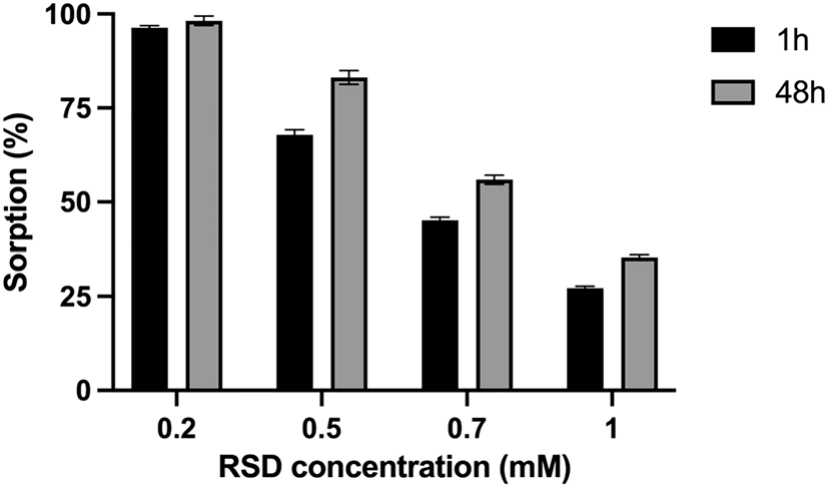
Sorption of risedronate (RSD) on hydroxyapatite at varying concentrations (0.2, 0.5, 0.7, and 1 mM) and time periods (1and 48 h), Data are presented in mean ± SE. N = 5. A two-way ANOVA was conducted to examine the effects of concentration (mM) and time (h) on the sorption of risedronate on hydroxyapatite. The Tukey's method was used for *post-hoc* comparison with a significance level of 0.05

**Fig. 2:**
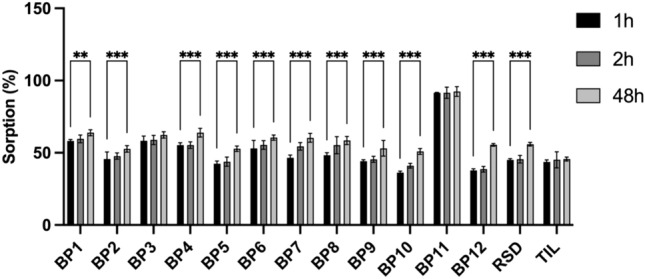
Sorption of various bisphosphonates (BP1 to BP12, RSD-risedronate, and TIL-tiludronate) on hydroxyapatite, measured at a concentration of 0.7 mM over different time periods (1, 2, and 48 h). Data are presented in mean ± SE. N = 5. A two-way ANOVA was conducted to examine the effects of BP and time (h) on the sorption values on hydroxyapatite. The Tukey's method was used for *post-hoc* comparison with a significance level of 0.05. Not significant comparisons with *p* values ≥ 0.05 are not labelled in the graphs

In our previous work, a series of analyses were performed to determine the affinity of the tested BPs to various materials simulating bone [14] and on this basis we selected the compounds for further researchand tpresented in this paper. The affinity of the RSD to HA was assessed under varying concentrations and time periods (Fig. 1). The sorption capacity was examined for 4 concentrations (0.2, 0.5, 0.7 and 1 mM). The effect of concentration and time on the sorption of RSD on HA was analyzed using a two-way ANOVA. The results indicated significant main effects for both concentration and time, as well as a significant interaction between these factors, effect of concentration (mM), F_3,32_ = 6658, p < 0.001p, effect of time (h), F_1,32_ = 654.7, p < 0.001, interaction effect (concentration × time), F_3,32_ = 63.22, p < 0.001. These results suggest that both the concentration of RSD and the duration of exposure significantly affect its sorption on HA, with higher concentrations resulting in reduced sorption capacity. Post-hoc comparisons using Tukey’s test confirmed these findings, showing significant differences between the tested concentrations and time points. For further investigation, the concentration of 0.7 mM was selected as it achieved approximately 50% RSD sorption. By increasing the concentration (compared to previous works [14]), it was possible to observe much higher sorption for several BPs compared to RSD (Fig. 2). A two-way ANOVA was conducted to examine the effects of BP type and time on their sorption values on HA. The results indicated significant main effects for both BP and time, as well as a significant interaction effect. Effect of BP, F_13,168_ = 289.6, p < 0.001, effect of time (h), F_2,168_ = 180.8, p < 0.001, interaction between BP and time, F_26,168_ = 4.987, p < 0.001. These findings indicate that the type of BP and the duration of exposure significantly affect sorption on HA, with notable interactions between these factors. Tukey’s post-hoc test confirmed significant differences among various BPs and time points. In one case, sorption occurred after just 1 h of contact with HA and did not increase with further exposure. This compound (BP11) showed the highest sorption, indicating the strongest affinity for HA. BP11 has a hydroxyl group on the pyridine ring, which is known to enhance HA binding [4]. Although none of the analyzed compounds had a hydroxyl group at the R1 site, they all had a secondary amine on the R2 site. BP1, which has no substituent in the pyridine ring and is similar to RSD, showed slightly higher affinity for HA than RSD. For BP2 to BP5, the presence and location of the methyl group varied, with BP3 and BP4 showing similar affinity to HA as BP1, while BP2 and BP5 exhibited lower affinity. BP6 and BP7, which contain iodine and bromine respectively, demonstrated slightly higher affinity for HA than RSD. Among the compounds containing chlorine in different positions, BP8 to BP10, only BP8 showed stronger affinity for HA than RSD. BP12, which features a benzothiazole group instead of a pyridine ring, displayed lower affinity for HA compared to RSD and BP1. These results suggest that the structural variations in BPs significantly influence their sorption capacity on HA.

### Parallel artificial membrane permeability assay (PAMPA)

As a class, BPs are hydrophilic drugs, which prevent diffusion across the gastrointestinal epithelium through the transcellular route. Lipophilic BPs are found to be far more active than any currently available BP drug [15]. The n-octanol–water partition coefficient is negative for hydrophilic substances. Zoledronate had poor permeability in the P. falciparum/red-cell assay due to its highly polar nature (log p = − 2.9) [16]. Log P and log D for RSD are also low- values in Table 4 were calculated using two programs and summarized. A two-way ANOVA was conducted to examine the effects of BP type and log type (log P and log D) on the calculated values. The results indicated significant main effects for both BP type and log type. Effect of BP, F_14,28_ = 15.51, p < 0.001, effect of log type, F_2, 28_ = 304.6, p < 0.001. Dunnett's multiple comparisons test was used for post-hoc comparison with a significance level of 0.05. The log P and log D values for the BPs, calculated using two different programs, are summarized in Table 4. The results show that all compounds (BP1-12), except for TIL, have higher log P and log D values compared to RSD. This means that RSD tends to have lower solubility in the nonpolar phase and lower distribution properties in biological conditions compared to these BPs. Therefore, it can be assumed that tested BPs will have higher permeability (compared to RSD). Especially BP11, BP12 and also BP6-BP9.

**Table 4:** Calculated log P and log D for various bisphosphonates (BP1 to BP12, RSD-risedronate, and TIL-tiludronate), A two-way ANOVA was conducted to examine the effects of bisphosphonate type and log type (Log P and Log D) on the values

BPs	Log P^a^	Log P^b^	Log D (pH 7)^b^	Adjusted *p* value vs. RSD
BP1	− 1.66	− 3.21	− 5.32	Ns
BP2	− 1.26	− 2.71	− 4.92	0.017
BP3	− 1.26	− 2.74	− 5.00	0.021
BP4	− 1.21	− 2.74	− 4.90	0.015
BP5	− 0.82	− 3.21	− 5.31	0.044
BP6	− 0.41	− 2.21	− 4.36	< 0.001
BP7	− 0.68	− 2.41	− 4.47	< 0.001
BP8	− 0.81	− 2.68	− 4.68	0.003
BP9	− 0.64	− 1.95	− 4.39	< 0.001
BP10	− 1.03	− 3.54	− 5.67	Ns
BP11	− 1.54	− 1.4	− 4.9	0.001
BP12	− 0.2	− 1.05	− 3.74	< 0.001
RSD	− 2.23	− 3.84	− 6.55	
TIL	0.49	0.91	− 3.37	< 0.001
Zoledronate	− 3.12	− 4.54	− 7.11	Ns

The results indicated significant main effects for both bisphosphonate type and log type. Dunnett's multiple comparisons test was used for post-hoc comparison with a significance level of 0.05

^a^Calculated with Molinspiration Cheminformatics.

^b^Calculated with Chemaxon

**Fig. 3:**
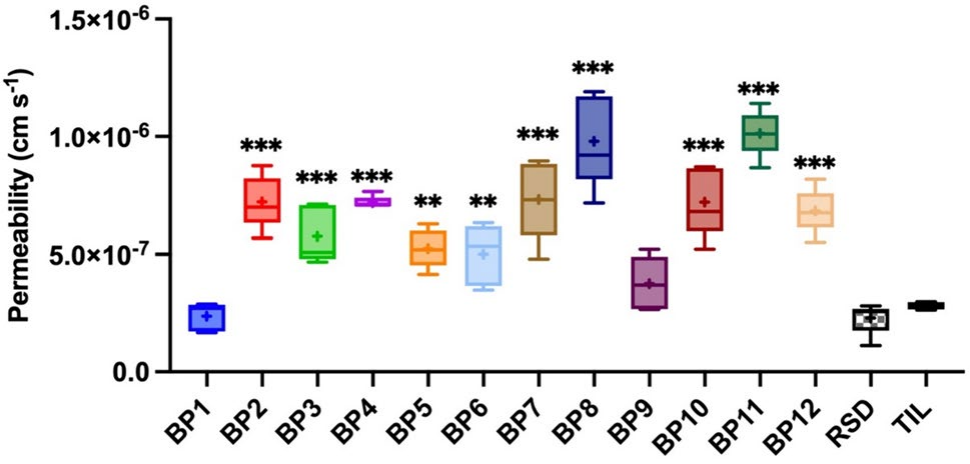
In vitro permeability of various bisphosphonates (BP1 to BP12), RSD (risedronate), and TIL (tiludronate) as reference compounds carried out using a parallel artificial membrane permeability assay (PAMPA). Whiskers represent the 10th to 90th percentiles, the box extends from the 25th to 75th percentiles, the midline is plotted at the median, and “ + ” is at the mean value, *N* = 5. The one-way ANOVA test revealed significant differences among the permeabilities of the various BPs (BP1 to BP12) and RSD. The Dunnett's multiple comparisons test was used for post-hoc comparison with a significance level of 0.05. Comparison of different BP to the RSD, statistical significance is indicated by symbols above each box, **p < 0.01, ***p < 0.001. Not significant comparisons with *p* values ≥ 0.05 are not labelled in the graphs. The colors in Figs are used to facilitate the assessment of changes for specific samples across different graphs

In vitro permeability tests showed that almost all tested BPs penetrated the PAMPA membrane more efficiently than RSD (Fig. 3). A one-way ANOVA revealed significant differences among the permeabilities of the various BPs (Effect of BP, F_13,56_ = 24, p < 0.001). The Dunnett's multiple comparisons test was used for post-hoc comparison with a significance level of 0.05. Ten out of twelve BPs (excluding BP1 and BP9) exhibited statistically significant differences in permeability compared to RSD. As we mentioned earlier, BP's oral bioavailability is low, reaching one percent- thus obtained permeability values are not high. The permeability of BP1 is similar to RSD (Fig. 3). The influence of the methyl group can be seen because all four compounds (BP2-BP5) penetrated the artificial membrane to a greater extent than BP1. The methyl group reduced the hydrophilicity of BPs, which is also visible from the calculated log P results. BPs with a methyl group in the *ortho* position (BP2) and *para* position (BP4) have higher permeability than the other two compounds. A significant difference can be seen between the two chlorine-containing compounds. BP8 permeability is higher than twice as much as BP9 permeability. Thus it can be seen that a slight difference in the position of the substituent can significantly affect the properties of the compound. Almost all tested BPs penetrated the PAMPA membrane more efficiently than RSD, the control compound. As shown in Fig. 3, ten out of twelve BPs tested (all except BP1 and BP9) exhibited statistically significant differences in permeability compared to RSD. Additionally, correlations can be seen between BPs with higher log P coefficients and higher permeability. When comparing the n-octanol-water partition coefficients of these two compounds, BP8 had a higher log P than BP9, further supporting this correlation.

### Bacterial cell response to BPs (BPs)

**Fig. 4:**
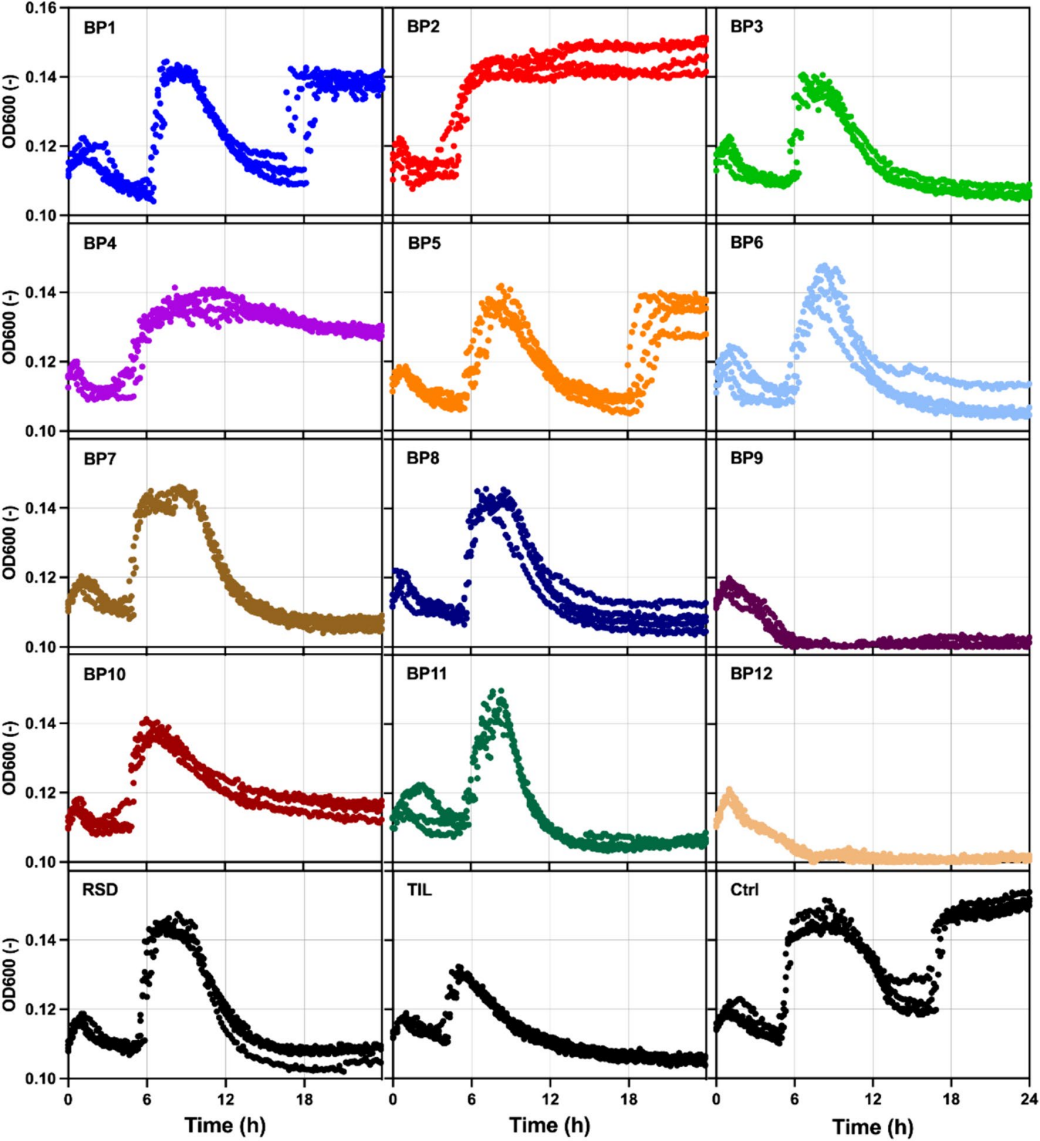
The growth curve of *L. paracasei* PCM 3039 in the absence (Ctrl) and presence of bisphosphonates (BP1-BP12, RSD-risedronate, TIL-tiludronate) was measured with a microplate reader at 36 °C. Each dot represents a separate experimental datapoint-data point. OD600—optical density at 600 nm

**Fig. 5:**
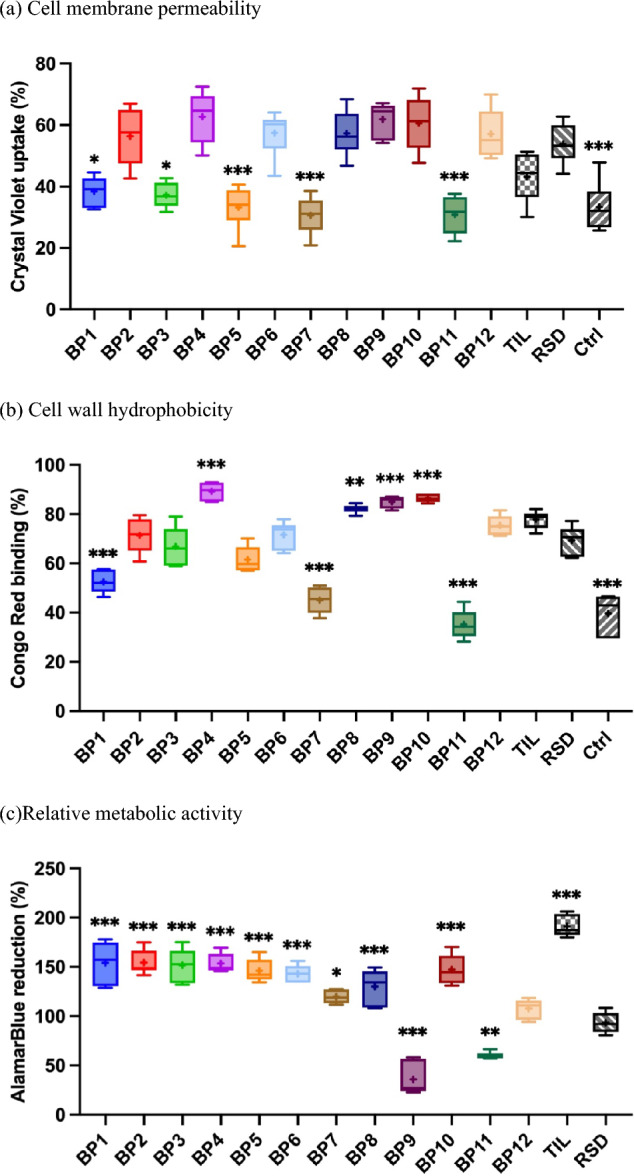
Comparison of cell properties of *L. paracasei* PCM 3039 in the absence (Ctrl) and presence of bisphosphonates (BP1-BP12, RSD-risedronate, TIL-tiludronate), cell membrane permeability (**a**), cell wall hydrophobicity (**b**), and relative metabolic activity (**c**). Relative metabolic activity of *L. paracasei* exposed to BPs is expressed as a percentage relative to the control group (100%). The control group represents untreated cells, and their metabolic activity was set as the baseline (100%) for comparison. The results for the control group were included in the calculations of the percentage reduction of AlamarBlue for the treated samples. Whiskers represent the 10th to 90th percentiles, the box extends from the 25th to 75th percentiles, the midline is plotted at the median, and “ + ” is at the mean value, *N* = 6. The results of the one-way ANOVA indicate a significant difference among the groups, cell membrane permeability, cell wall hydrophobicity and relative metabolic activity. The Tukey's method was used for *post-hoc* comparison with a significance level of 0.05. Comparison of different BP to the RSD, statistical significance is indicated by symbols above each box, *p < 0.05, **p < 0.01, ***p < 0.001. Not significant comparisons with *p* values ≥ 0.05 are not labelled in the graphs

During experiments associated with lactic acid bacteria, the growth of *L. paracasei* in the presence and absence of BPs was monitored (Fig. 4). Modifications of the cell surface properties were also evaluated (Fig. 5).

As presented in Fig. 4, the shape of a bacterial growth curve differed, depending on the source of carbon used in the culture. Reference cells in the Ctrl culture started dividing regularly in about five hours. During the sixth hour of incubation, they entered the stationary phase which last about four—five hours. Next, the cell count decreased drastically. Afterwards, the cells started to multiply again and after 16–17 h entered another stationary phase. A similar shape of the growth curve was observed for cells from the BP1 and BP5 cultures, however, their growth was about one hour slower, and the cell density was lower. In the cultures containing BP3, BP6, BP7, BP8, BP10, BP11, and RSD, the initial fast cell growth was followed by a rapid decrease in cell density. In these cultures, the cells did not start to grow again until the 24th hour of incubation. The measurements can provide important information as lengths of specific growth phases can act as indicators of cellular stress [34]. In addition, the modifications of the shape of the bacterial growth curve reflect the regulation of numerous metabolic processes occurring within the cells [35].

In general, the best growth, manifested by the highest OD_600_ and fast adjustment to the new environment, was observed for the cultures containing BP2. A similar shape of the growth curve was noted for culture containing BP4, however, the cell density reached was significantly lower. Cell growth was significantly inhibited in the presence of BP9 and BP12. This can be indicative that these two compounds showed the greatest toxicity towards *L. paracasei.*

The modifications of the Crystal Violet uptake, Congo Red binding, and relative cell metabolic activity (Fig. 5) were measured to analyze the impact of BPs with different substituents on *L. paracasei* one of the most important representatives of lactic acid bacteria (LAB) that colonizes the human gut [36].

To analyze the impact of selected BPs on the cells of *L. paracasei*, three tests were carried out based on spectrophotometric measurements of samples after the color change of the reagents used. The results of the one-way ANOVA indicate a significant difference among the groups, cell membrane permeability F_14,75_ = 18.2, p < 0.001, cell wall hydrophobicity F_14,75_ = 63.5, p < 0.001, relative metabolic activity F_13,70_ = 60.8, p < 0.001. The Tukey's method was used for *post-hoc* comparison with a significance level of 0.05. In the first stage, changes in the total permeability of cell membranes were examined. As shown in Fig. 5-a, seven out of twelve BPs tested (namely BP2, BP4, BP6, BP8, BP9, BP10, BP12) caused statistically significant changes in Crystal Violet uptake compared to the reference cells (Ctrl) (33.4 ± 8.0%). Exposure of bacterial cells to all BPs increased the permeability of cell membranes. Cells exposed to BP10, BP9, and BP4 reached the highest values of membrane permeability. They were 60.6 ± 8.7%, 61.9 ± 5.7%, and 62.7 ± 8.4%, respectively. Comparing aminomethyleneBPs with a sample containing standard RSD, can be observed that the exposure of bacterial cells to the newly synthesized compounds, resulted in a decrease in the permeability of cell membranes (in all statistically significant samples). The lowest Crystal Violet uptake values were 30.9 ± 6.4% (BP11) and 30.6 ± 6.0% (BP7). The test with the use of Congo Red allowed us to observe changes in the binding of the Congo Red dye, which indicates changes in cell surface hydrophobicity. This, however, can be indicative of the changed propensity of microorganisms to adhesion and remodeling of the outer structures of the cell [37]. As can be seen in Fig. 5b, the direction of changes in the hydrophobicity of cells is very similar to that obtained during the analysis of cell membrane permeability. The Congo Red Binding values obtained for all tested samples increased compared to the Ctrl sample (39.7 ± 8.0%). The highest hydrophobicity values were obtained for samples BP8, BP9, BP10, BP4. They were 82.1 ± 1.7%, 85.0 ± 2.4%, 86.4 ± 1.7% and 89.3 ± 3.5%, respectively. These BP samples were also the only ones where an increase in hydrophobicity of cells was observed compared to the RSD sample. The lowest Congo Red Binding values were 45.0 ± 5.8% (BP7) and 35.2 ± 6.1% (BP11).

The changes in cell membrane permeability and cell hydrophobicity may be closely related to cellular stress, detoxification of cells, or protection from a toxic environment. Therefore, the next stage of the microbial analysis of the impact of BPs on *L. paracasei* was to study their cytotoxicity.

The toxicity results of selected DILs are shown as the percent reduction of the AlamarBlue test reagent—Fig. 5c. The metabolic activity of *Lactobacillus paracasei* cells exposed to BPs was evaluated and expressed as a percentage relative to the control group, which was set at 100%. This control group represents the baseline metabolic activity of untreated cells. The greater the reduction, the lower the toxicity of the tested compounds. Cells have adapted to the presence of almost all tested BPs which were not harmful for them. Only for BP11 and BP9 samples, the decrease in cell viability was observed. This result is also clearly visible in Fig. 4, which shows the growth curves of bacterial cells. The AlamarBlue Reduction values for these samples were 60.4 ± 3.4% and 35.9 ± 16.7%, respectively. These BP samples were also the only ones where a decrease in toxicity of tested compounds was observed, compared to the RSD sample. The highest reduction values were obtained for BP1, BP2, and BP4 samples, and they were approximately 154.0%.

An exposure of *L. paracasei* to various BPs had a significant effect on the tested parameters. The obtained results indicate that changes in cell membrane permeability and cell surface hydrophobicity are strongly related and dependent on the toxicity of the tested compounds. Moreover, the observed changes may be closely related to the structure of the selected aminomethyleneBPs. Particular changes have occurred in several samples differing in the presence of other functional groups. These samples are especially, BP4—where in the structure of the compound a methyl group is substituted in the *para* position, BP8, BP9, BP10—where in the structure of the compounds a chlorine is substituted in the *para, meta,* and *ortho* position, respectively, BP11—where in the structure of the compound a hydroxyl group is substituted in the *meta* position. An increase in cell membrane permeability and cell surface hydrophobicity compared to the Ctrl sample also corresponds to an increase in cell viability. However, a decrease in these parameters is responsible for greater toxicity of BPs. It can be concluded that the bacterial cells under the influence of the presence of BP4, BP8, and BP10 tried to detoxify their environment, which led to a significant increase in bacterial viability. The opposite situation was observed for samples BP9 and BP11.

The results of all experiments indicate that each of the compounds tested has influenced *L. paracasei* cells differently. Regarding the membrane permeability, the significant decrease was observed for cultures containing BP1, BP3, BP5, BP7, and BP11 in comparison with RSD. Importantly, all these samples were not statistically different from the reference cells (Ctrl). Modifications of the Congo Red binging indicate strong remodeling of the bacterial cell wall in comparison with both, RSD and Ctrl.

## Discussion

We tested a series of aminobisphosphonates and identified several compounds with more favorable properties than those of RSD based on three different studies. Initially, we determined the compounds with higher affinity to HA, indicating stronger therapeutic potential. Seven compounds showed a higher affinity for HA than RSD. The highest sorption degree was obtained for BP11, which has a hydroxyl group at the pyridine ring. Variations in mineral affinity likely stem from the orientation of their R2 nitrogen atoms facilitating N-H-O bond formation at crystal surfaces. Modeling studies revealed that optimal binding entails a bond angle ≥ 125° and a bond distance of 3 Å. In contrast, RSD exhibited a 3 Å N-O distance and only a 102° bond angle [13].

The binding of BPs to bone surfaces involves a physicochemical reversible process, allowing for release through chemical desorption or osteoclastic resorption mechanisms. For instance, the lower-affinity BP, clodronate, demonstrates greater diffusion through bone compared to etidronate due to enhanced desorption properties [3]. In our prior work, we selected BPs that not only sorbed more strongly than RSD but also desorbed rapidly [14]. Specifically, compounds BP1, BP2, BP6-BP9, and BP12 exhibited high sorption levels on polymer-ceramic materials, followed by extensive desorption—a desired trait for potential antiosteoporotic agents.

Subsequently, we assessed which compounds showed improved penetration of the artificial intestine, indicating greater bioavailability. BP2, BP4, BP7, BP8, and BP10-BP12 displayed enhanced permeability through the artificial membrane, with BP8 and BP11 exhibiting the highest permeability among all analyzed BPs. The drug concentration of BP2, BP4, BP7, BP8, and BP10-BP12 in the acceptor plate was over twice as high as RSD, suggesting enhanced bioavailability (Fig. 3). A higher amount of the drug permeating through the artificial membrane would have a beneficial therapeutic effect if only BPs will cause healing properties. BP11 showed the strongest affinity for HA. Of the other compounds listed above (as those with high permeability), three also showed a strong affinity for bone: BP4, BP7, and BP8.

The relative metabolic activity of cells exposed to the newly synthesized BPs was higher than that of RSD. The only exceptions are BP9, BP11, and BP12 for which a decrease (BP9, BP11) or no statistically significant change (BP12) was observed. In addition, BP9 and BP12 strongly inhibited the bacterial growth rate. This indicates that these compounds have the strongest negative effect on the bacterial cells. The rest of the BPs, especially BP1, and BP5, can be considered as relatively mild compounds that do not disturb the beneficial bacterial cells, colonizing the human gut. Taking into account all the obtained results, several compounds with more favorable properties than the commercially used RSD can be identified, these are BP3, BP4, BP6-8, and BP11 and it is worth investigating them further. Although our findings suggest a potential interaction between BPs and lactic acid bacteria, we acknowledge that the gastrointestinal side effects of BPs are multifactorial. Therefore, while our study provides valuable insights into one aspect of BP interaction, further research is needed to comprehensively understand their broader impacts on gut health.

The observed increase in cell membrane permeability in response to BPs can be linked to their interaction with the phospholipid bilayer of bacterial cells. BPs, particularly those with higher lipophilicity, can integrate into the lipid bilayer, causing disruptions. This phenomenon has been documented in studies examining the interaction of hydrophilic and lipophilic compounds with cell membranes [10, 16]. These studies highlight how changes in lipophilicity affect membrane permeability and compound transport across cell membranes. The modifications in cell wall hydrophobicity and permeability are indicative of cellular responses to toxic substances, which may include the activation of detoxification mechanisms. These mechanisms often involve alterations in membrane structure and function to mitigate the toxic effects of external compounds. The increase in hydrophobicity observed in our study suggests an adaptive response by *Lactobacillus paracasei* to maintain cell integrity in the presence of BPs. Similar responses have been reported in studies exploring bacterial adaptation to environmental stressors [34, 37].

Following the study on BPs, it is notable that several tested compounds exhibited potentially beneficial therapeutic properties compared to commonly used BPs. Compounds with stronger affinity for bone and enhanced gastrointestinal absorption were identified, suggesting their potential therapeutic efficacy.

Based on the results obtained, it can be concluded that the newly synthesized BPs may serve as promising candidates for further exploration of potential therapeutic applications.

The investigated BPs displayed varying levels of toxicity towards bacterial cells, emphasizing the need for further investigation into their impacts on bacterial flora.

The findings from this study set the stage for further exploration into BPs, including delving into their potential mechanisms of action and developing more targeted therapies.

## Data Availability

The datasets generated during and/or analysed during the current study are available from the corresponding author upon reasonable request.

## Abbreviations

BPs: Bisphosphonates
HA: Hydroxyapatite
PAMPA: Parallel artificial membrane permeability assay
PBS: Phosphate-buffered saline
RSD: Risedronate
TIL: Tiludronate

## References

[1] Pouresmaeili F, Kamali Dehghan B, Kamarehei M, Yong MG. A comprehensive overview on osteoporosis and its risk factors. Ther Clin Risk Manag. 2018;14:2029–49. 10.2147/TCRM.S138000. 30464484 10.2147/TCRM.S138000 PMC6225907

[2] Clunie G, Keen RW. Osteoporosis. 2nd ed. Oxford: Oxford University Press; 2014.

[3] Ebetino FH, Rozé CN, McKenna CE, Barnett BL, Dunford JE, Russell RGG, et al. Molecular interactions of nitrogen-containing bisphosphonates within farnesyl diphosphate synthase. J Organomet Chem. 2005;690:2679–87. 10.1016/j.jorganchem.2005.03.005. 10.1016/j.jorganchem.2005.03.005

[4] Russell RGG, Xia Z, Dunford JE, Oppermann U, Kwaasi A, Hulley PA, et al. Bisphosphonates: an update on mechanisms of action and how these relate to clinical efficacy. Ann N Y Acad Sci. 2007;1117:209–57. 10.1196/annals.1402.089. 18056045 10.1196/annals.1402.089

[5] Petneházy I, Jászay ZM, Töke L. Phosphite addition to carbonyl group and phosphoryl migration under phase transfer catalytic circumstances. Phosphorus Sulfur Silicon Relat Elem. 1996;109:421–4. 10.1080/10426509608545180. 10.1080/10426509608545180

[6] McClung MR, Ebetino FH. History of risedronate. Bone. 2020. 10.1016/j.bone.2020.115407. 32387834 10.1016/j.bone.2020.115407

[7] Zielińska M, Garbacz G, Sczodrok J, Voelkel A. The effects of various food products on bisphosphonate’s availability. Pharmaceutics. 2022;14:717. 10.3390/pharmaceutics14040717. 35456551 10.3390/pharmaceutics14040717 PMC9029784

[8] Ghosh S, Chan JMW, Lea CR, Meints GA, Lewis JC, Tovian ZS, et al. Effects of bisphosphonates on the growth of Entamoeba Histolytica and plasmodium species in vitro and in vivo. J Med Chem. 2004;47:175–87. 10.1021/jm030084x. 14695831 10.1021/jm030084x

[9] Jeon O-C, Seo D-H, Kim H-S, Byun Y, Park JW. Oral delivery of zoledronic acid by non-covalent conjugation with lysine-deoxycholic acid: In vitro characterization and in vivo anti-osteoporotic efficacy in ovariectomized rats. Eur J Pharm Sci. 2016;82:1–10. 10.1016/j.ejps.2015.11.004. 26542347 10.1016/j.ejps.2015.11.004

[10] Kansy M, Senner F, Gubernator K. Physicochemical high throughput screening: parallel artificial membrane permeation assay in the description of passive absorption processes. J Med Chem. 1998;41:1007–10. 10.1021/jm970530e. 9544199 10.1021/jm970530e

[11] Di L, Kerns EH, Fan K, McConnell OJ, Carter GT. High throughput artificial membrane permeability assay for blood–brain barrier. Eur J Med Chem. 2003;38:223–32. 10.1016/S0223-5234(03)00012-6. 12667689 10.1016/S0223-5234(03)00012-6

[12] Oktabec Z, Kos J, Mandelova Z, Havelkova L, Pekarek T, Rezacova A, et al. preparation and properties of new co-crystals of ibandronate with gluco- or galactopyranoside derivatives. Molecules. 2010;15:8973–87. 10.3390/molecules15128973. 21150819 10.3390/molecules15128973 PMC6259308

[13] Russell RGG, Watts NB, Ebetino FH, Rogers MJ. Mechanisms of action of bisphosphonates: similarities and differences and their potential influence on clinical efficacy. Osteoporos Int. 2008;19:733–59. 10.1007/s00198-007-0540-8. 18214569 10.1007/s00198-007-0540-8

[14] Zielińska M, Chmielewska E, Buchwald T, Voelkel A, Kafarski P. Determination of bisphosphonates anti-resorptive properties based on three forms of ceramic materials: sorption and release process evaluation. J Pharm Anal. 2021;11:364–73. 10.1016/j.jpha.2020.07.011. 34277124 10.1016/j.jpha.2020.07.011 PMC8264463

[15] Zhang Y, Cao R, Yin F, Hudock MP, Guo R-T, Krysiak K, et al. Lipophilic bisphosphonates as dual farnesyl/geranylgeranyl diphosphate synthase inhibitors: An X-ray and NMR investigation. J Am Chem Soc. 2009;131:5153–62. 10.1021/ja808285e. 19309137 10.1021/ja808285e PMC2753403

[16] No JH, De Macedo DF, Zhang Y, Liu Y-L, Zhu W, Feng X, et al. Lipophilic analogs of zoledronate and risedronate inhibit Plasmodium geranylgeranyl diphosphate synthase (GGPPS) and exhibit potent antimalarial activity. Proc Natl Acad Sci U S A. 2012;109:4058–63. 10.1073/pnas.1118215109. 22392982 10.1073/pnas.1118215109 PMC3306666

[17] Liu J, Curtis EM, Cooper C, Harvey NC. State of the art in osteoporosis risk assessment and treatment. J Endocrinol Invest. 2019;42:1149–64. 10.1007/s40618-019-01041-6. 30980341 10.1007/s40618-019-01041-6 PMC6751157

[18] Compston JE, McClung MR, Leslie WD. Osteoporosis. Lancet. 2019;393:364–76. 10.1016/S0140-6736(18)32112-3. 30696576 10.1016/S0140-6736(18)32112-3

[19] Hove H, Nørgaard H, Brøbech MP. Lactic acid bacteria and the human gastrointestinal tract. Eur J Clin Nutr. 1999;53:339–50. 10.1038/sj.ejcn.1600773. 10369488 10.1038/sj.ejcn.1600773

[20] Rattanaprasert M, Roos S, Hutkins RW, Walter J. Quantitative evaluation of synbiotic strategies to improve persistence and metabolic activity of Lactobacillus reuteri DSM 17938 in the human gastrointestinal tract. J Funct Foods. 2014;10:85–94. 10.1016/j.jff.2014.05.017. 10.1016/j.jff.2014.05.017

[21] Vaughan EE, Heilig HGHJ, Ben-Amor K, De Vos WM. Diversity, vitality and activities of intestinal lactic acid bacteria and bifidobacteria assessed by molecular approaches. FEMS Microbiol Rev. 2005;29:477–90. 10.1016/j.fmrre.2005.04.009. 16125009 10.1016/j.fmrre.2005.04.009

[22] Li X, Liu L, Cao Z, Li W, Li H, Lu C, et al. Gut microbiota as an “invisible organ” that modulates the function of drugs. Biomed Pharmacother. 2020. 10.1016/j.biopha.2019.109653. 31810138 10.1016/j.biopha.2019.109653

[23] Sirichoat A, Flórez AB, Vázquez L, Buppasiri P, Panya M, Lulitanond V, et al. Antibiotic susceptibility profiles of lactic acid bacteria from the human vagina and genetic basis of acquired resistances. Int J Mol Sci. 2020;21:2594. 10.3390/ijms21072594. 32276519 10.3390/ijms21072594 PMC7178285

[24] Wei F, Zhou L, Wang Q, Zheng G, Su S. Effect of compound lactic acid bacteria capsules on the small intestinal bacterial overgrowth in patients with depression and diabetes: a blinded randomized controlled clinical trial. Dis Markers. 2022;2022:1–6. 10.1155/2022/6721695. 10.1155/2022/6721695 PMC9168214 35677636

[25] Matczak-Jon E, Ślepokura K, Kafarski P. Solid state and solution behaviour of N-(2-pyridyl)- and N-(4-methyl-2-pyridyl)aminomethane-1,1-diphosphonic acids. J Mol Struct. 2006;782:81–93. 10.1016/j.molstruc.2005.07.004. 10.1016/j.molstruc.2005.07.004

[26] Matczak-Jon E, Sawka-Dobrowolska W, Kafarski P, Videnova-Adrabinska V. Molecular organization and solution properties of N-substituted aminomethane-1,1-diphosphonic acids. New J Chem. 2001;25:1447–57. 10.1039/b102282m. 10.1039/b102282m

[27] Matczak-Jon E, Ślepokura K, Kafarski P. [(5-Bromopyridinium-2-ylamino)- (phosphono)methyl]phosphonate. Acta Crystallogr C. 2006;C62:o132–5. 10.1107/S0108270106002423 16518048

[28] Matczak-Jon E, Ślepokura K. Conformations and resulting hydrogen-bonded networks of hydrogen {phosphono[(pyridin-1-ium-3-yl)amino]methyl}phosphonate and related 2-chloro and 6-chloro derivatives. Acta Crystallogr C. 2011;C67:o450–6. 10.1107/S0108270111040650. 10.1107/S0108270111040650 22051961

[29] Widler L, Jaeggi KA, Glatt M, Müller K, Bachmann R, Bisping M, et al. Highly potent geminal bisphosphonates. From pamidronate disodium (aredia) to zoledronic acid (zometa). J Med Chem. 2002;45:3721–38. 10.1021/jm020819i. 12166945 10.1021/jm020819i

[30] Pietrzyńska M, Voelkel A. Stability of simulated body fluids such as blood plasma, artificial urine and artificial saliva. Microchem J. 2017;134:197–201. 10.1016/j.microc.2017.06.004. 10.1016/j.microc.2017.06.004

[31] Krishnamurthi VR, Niyonshuti II, Chen J, Wang Y. A new analysis method for evaluating bacterial growth with microplate readers. PLoS ONE. 2021. 10.1371/journal.pone.0245205. 33434196 10.1371/journal.pone.0245205 PMC7802944

[32] Pacholak A, Burlaga N, Guzik U, Kaczorek E. Investigation of the bacterial cell envelope nanomechanical properties after long-term exposure to nitrofurans. J Hazard Mater. 2021. 10.1016/j.jhazmat.2020.124352. 33160784 10.1016/j.jhazmat.2020.124352

[33] Pacholak A, Burlaga N, Frankowski R, Zgoła-Grześkowiak A, Kaczorek E. Azole fungicides: (Bio)degradation, transformation products and toxicity elucidation. Sci Total Environ. 2022. 10.1016/j.scitotenv.2021.149917. 34525765 10.1016/j.scitotenv.2021.149917

[34] Hamill PG, Stevenson A, McMullan PE, Williams JP, Lewis ADR, Gillion JL, et al. Microbial lag phase can be indicative of, or independent from, cellular stress. Sci Rep. 2020;10:5948. 10.1038/s41598-020-62552-4. 32246056 10.1038/s41598-020-62552-4 PMC7125082

[35] Peleg M, Corradini MG. Microbial growth curves: what the models tell us and what they cannot. Crit Rev Food Sci Nutr. 2011;51:917–45. 10.1080/10408398.2011.570463. 21955092 10.1080/10408398.2011.570463

[36] Foysal MJ, Fotedar R, Siddik MAB, Tay A. Lactobacillus acidophilus and L. plantarum improve health status, modulate gut microbiota and innate immune response of marron (Cherax cainii). Sci Rep. 2020;10:5916. 10.1038/s41598-020-62655-y. 32246011 10.1038/s41598-020-62655-y PMC7125160

[37] Krasowska A, Sigler K. How microorganisms use hydrophobicity and what does this mean for human needs? Front Cell Infect Microbiol. 2014;4:112. 10.3389/fcimb.2014.00112. 25191645 10.3389/fcimb.2014.00112 PMC4137226

